# You've Got to Be Real: Authenticity, Performativity and Micro-Celebrity in South Africa

**DOI:** 10.3389/fsoc.2021.652485

**Published:** 2021-05-07

**Authors:** Callan Dunn, Nicky Falkof

**Affiliations:** Department of Media Studies, University of the Witwatersrand, Johannesburg, South Africa

**Keywords:** Instagram, post-feminism, South Africa, social media, micro-celebrities

## Abstract

For many young black South African women, the competitive arena of social media offers access to significant social and cultural capital, which can be invaluable in the unequal context in which they live. In order to succeed in this high stakes environment young women carefully construct the identities and idealised selves that they present on platforms like Instagram. They display a lifestyle of glamorous consumption, showcasing exclusive brands and fashionable items and modifying and modelling themselves to fit a beauty ideal that emphasises youth, light skin, slender bodies and straight hair. As well as these physical features, young women on Instagram are also hyper-aware of the need to appear “authentic”: to have their online lives and selves appear natural, easy and free of artifice in order to further enhance their status as role models to other women. This article draws from in-depth interviews with 10 black South African “micro-celebrities.” It reveals the central role of authenticity in these young women's online performances of self, and considers the contradictory impulses that require them to both “feel” and “appear” real. Within the framework of existing hegemonic structures, these women appear to be exercising their freedom as neoliberal citizens within a post-feminist setting. Despite the promises of freedom, however, this article reveals the way in which their performances of selfhood are powerfully constrained by normative ideas about aspiration and success.

For many young black South African women the competitive arena of social media offers access to social and cultural capital that can be difficult to come by in a context of deep inequality, with poverty, economic uncertainty and stagnating class mobility as persistent features of the post-apartheid racial order. In order to acquire a large number of followers, thus making themselves attractive to high status romantic partners and to brands and marketing companies who will help them to monetise their personas, these “micro-celebrities” carefully construct idealised selves that they present on platforms like Instagram. They engage in particular beauty practises, presenting a version of selfhood that is aspirational/inspirational and, at the same time, appears “authentic”: genuine, relatable, unvarnished and honest. In order to succeed in the high stakes environment of Instagram, these young women must appear both unattainably glamorous and approachably real. Their personas emphasise freedom and agency, but their freedom and agency are constrained by normative expectations about character, appearance and even morality.

This article is focused on 10 black South African micro-celebrities, each aged between 18 and 25, each with over 10,000 followers. Marwick defines micro-celebrity as “a mind-set and a collection of self-presentation practices endemic in social media, in which users strategically formulate a profile, reach out to followers, and reveal personal information to increase attention and thus improve their online status” (2015, p. 139). Micro-celebrities are an increasingly visible phenomenon in South Africa, sometimes leveraging their digital personas to access significant social and economic capital (see Iqani, 2019). They also straddle the gap between ordinary social media users and “legitimate” celebrities, relying on both the authenticity of the everyday and the glamour of the exceptional to create their public profiles.

All of the women in this study subscribe to a particular look, characterised by slender bodies, long well-groomed hair (weaves), light skin, immaculate clothing and desirable accessories. They pride themselves on paying close attention to their physical appearances. We discuss in-depth interviews with all 10 in order to examine how the idea of authenticity impacts on their online and offline lives and the way in which they construct their digital personas.^1^ We argue that their definition of “real” is not real in itself; rather it is a performance of realness and authenticity that is carefully constructed within the constraints of societal norms and expectations, and then displayed as another commodity in an arsenal of appealing and aspirational traits.

Social media allow people to express themselves, define and re-define who they are, and curate who they would like to be within the online space (Schau and Gilly, 2003). By participating in social media platforms, black South African women, like other users, “adventurously [harness] them as vehicles for identity and identification” (De Bruijn and Nyamnjoh, 2009, p. 14). Particularly because of its photo-based format, Instagram allows users to “develop their self-concept and affiliated identities to create their image, and to produce their own spotlight through the experience” (Pugh, 2010, p. 1). The aesthetics involved in this aspirational performance provide an opportunity “to create a desirable image of life where every experience is visualized” (Lindahl and Öhlund, 2013, p. 6). In the case of these South African micro-celebrities, an aesthetics of aspiration is supplemented by a visual and verbal discourse of authenticity.

In its most common use, authenticity is connected to the idea of expressing one's individuality through personalised traits and acting in accordance with one's own genuine beliefs and desires. This view of authenticity can extend to “the idea that some things are in some sense really you, or express what you are, and others aren't” (Williams, 2002, p. 277). Authenticity emphasises the humanness of the individual, a humanness that is “innate, essence-like (deep-seated and fundamental), cross-culturally universal, and typical of the human population” (Haslam et al., 2011, p. 207). Additionally, to be authentic “is to be clear about one's own most basic feelings, desires and convictions, and to openly express one's stance the public arena” (Guignon, 2008, p. 288). These technical definitions of authenticity do not, however, account for its status as a cultural commodity, which in turn means that authenticity can be claimed, performed, adopted or exaggerated for social capital. As emphasised by Salisbury and Pooley (2017, p. 2), “authentic is always relative to something else, and is therefore susceptible to the charge of phoniness, especially if strategy or calculation can be identified.” In contrast to utopian notions of authenticity, which suggest that there is an unmediated “real” self that can be accessed and shared, these perspectives suggest that the self is always to some extent socially and culturally constructed, whether online or offline. Such an understanding is valuable when considering young women whose everyday bodily aesthetics are often subject to, or formed in resistance to, prevailing social (and social media) norms. Rather than asking whether such performances are “genuinely” authentic, we need to consider what the *idea* of authenticity means, how it is marketised and how it is used to maintain a sense of identity under conditions of networked performativity.

Establishing a style and sustaining it on social media is one of the ways in which people display their “real” versions of self in the public arena (Varga, 2011). Appearing authentic in public involves being confident or open enough to speak in a personal way, using an informal understandable tone and sharing personal content and details about one's life. This approach gives observers and followers the sense that one is truthful about what one stands for, because of the consistency in sharing the “real” aspects of who one is in a visible way, as people are given some degree of access to the “real” person (Gaden and Dumitrica, 2015).

Part of the appeal of social media is the idea that sharing intimate information about one's life leads to the development of stronger social bonds (John, 2013). This apparent intimacy can be considered in terms of what Dean (2002) calls the “ideology of publicity,” in which individuals have identified what others want to see and act accordingly. This representation of the self is not a rigid construct that develops in isolation, but is instead the result of the interactions of a variety of “social and cultural categories and identifications” (Harris, 2004, p. 3). Thus, the process that people go through to be acknowledged by others involves the identification and endorsement of the “authentic” individual by the audience in the public-private arena that is facilitated by social media (Papacharissi, 2010).

## A Post-Feminist Paradox

It is useful to consider these complexities in terms of post-feminism, which can be summarised as a set of popular discourses claiming that feminism is no longer necessary as women can now become empowered through shopping, bodily enhancement and modification, career success and assuming a prominent position in a male-dominated society (McRobbie, 2007). The connotations and expectations that surround women in a post-feminist era are defined around “capacity, success, attainment, enjoyment, entitlement, social mobility and participation” (Giddens, 1991, p. 46).

Much of the most influential scholarship on post-feminism focuses on its relations to consumption, beauty and the body. Post-feminist bodies are observed, disciplined and trained into “appropriate” modes of being that are seen to quite literally embody the individual's freedom, agency and empowerment. Because the female body “possesses power, and is in constant need of monitoring, surveillance and re-modelling” (Gill, 2007b, p. 441), the post-feminist subject views the “self as a project” (Tasker and Negra, 2007, p. 21). Indeed, post-feminism “promises women that their “capital” lies especially in beauty” (Dosekun, 2017, p. 179). The disciplined body is also a consumerist body, one that purchases and displays clothing, products and consumer items that are posited as desirable or even essential. According to McRobbie (2009, p. 27), the domain of “leisure and consumer culture is dominated by the vocabulary of personal choice, and is a primary site for hedonism, fantasy, personal gratification and entertainment.” This means that post-feminist performance is a function of class, as only women with the necessary means are able to access this type of consumerism. In South Africa, a hugely unequal country where economic consequences of the racialised system of apartheid still persist, upwardly mobile young black women must often find creative ways to enter into consumer culture. These may include what Masango calls “compensated relationships” (Masango, 2019, p. 8), as well as the kind of social media self-branding that characterises the micro-influencers in this study.

Post-feminism is also intimately related to neo-liberalism, a mode of thought that emphasises choice and individuality, often co-opted from the language of progressive politics (McRobbie, 2009). According to Gill, “Neoliberalism is increasingly understood as constructing individuals as entrepreneurial actors who are rational, calculating and self-regulating” (Gill, 2008, p. 443). The neo-liberal woman is imagined as autonomous and self-analysing, and has the freedom of choice and agency to self-modify (Gill, 2007a).

This emphasis on individuality, agency and autonomy is part of the paradox of post-feminism: a relation to femininity that rejects collective politics in favour of the choice and personal success of the individual, but with this choice and personal success framed in ways that require enthusiastic consumption and bodily management. The post-feminist and neo-liberal valorisation of the idea of the individual and her empowered agency feeds directly in to the inflated valuation of the notion of authenticity. Post-feminism involves “an emphasis on empowerment and individualism” (Gill, 2007b). In order to be a successful post-feminist subject, and a successful Instagram personality, one must display *authentic* individuality. One must be seen to remain “true to oneself” even while engaging in a performance of femininity that is coherent with normative ideas about attractiveness, style, behavior and morality. Authenticity thus becomes another pole of post-feminist praxis, one that is as intimately related to the self as the body is.

Drawing on the psychoanalytic theory of Joan Riviere, we can think of this as a kind of masquerade. Like femininity itself, authenticity is “not natural, but an adopted surface, a defence mechanism” (Riviere, 1929, p. 306). For women whose digital personae are formed within a post-feminist milieu, their self-portrayal is not as straightforward as it seems, which again highlights the underlying political/patriarchal power that instructs this apparently autonomous behavior (McRobbie, 2007). Through this masquerade, which encompasses both physical work and the social-emotional labour of performing authenticity, women can achieve a sense of acceptance through “mimicry, accommodation, adjustment and modification” (McRobbie, 2009, p. 71). Literature on post-feminism reveals that these modes of self-display are not about what a woman “naturally is,” or about her freedom from social constraint, but rather about how women replicate specific ideas about femininity.

While much of the canonical literature on post-feminism is located in the global north, important scholarship from Dosekun highlights the “transnational” qualities of post-feminism Dosekun (2015), the way it is “broadcast and sold across borders [and] interpellates subject-consumers that have the material, discursive and imaginative capital to buy into it, their diverse locations and histories notwithstanding” (2017, p. 169). The young women in our study illustrate the globalised power of certain displays of femininity which transcend borders and boundaries and settle comfortably within the African context. Rather than discussing themselves in terms of South Africa or Africa, they look to global and African-American celebrities like Beyonce and Rihanna for their inspiration and aspiration. They also, however, present a particular case study of how post-feminist ideas manifest in Africa. Their online gender performances, and the motivations behind them, are inflected by South African aesthetics as well as ideas about race, status, society and female acceptability.

## Methods and Participants

This project adopts a qualitative approach involving a “detailed examinations of cases that arise in the natural flow of social life” (Neuman, 2006, p. 14). It is concerned with “developing explanations of social phenomena, and aims to help us understand the social world in which we live and why things are the way they are” (Hancock et al., 2009, p. 7). This approach allows room for interpretations of the world and a more flexible acquisition of knowledge (Stokes, 2003).

During an initial period of digital research we observed a number of active Instagrammers who fitted our profile, whose images were readily observable within the public domain and not hampered by restrictions, except for an active Instagram account. We approached some of them via the direct message feature and used snowball sampling to recruit other participants. This method allowed us to “take advantage of the social networks of identified respondents to provide a researcher with an ever-expanding set of potential contacts” (Atkinson and Flint, 2001, p. 1). After receiving appropriate ethics clearance from our institution, we scheduled interviews with each willing participant. Each was approximately one and a half hours in length, and took the form of either a Skype or Facetime call. These semi-structured qualitative interviews were conducted in 2016. Semi-structured interviews recognise common themes, which contribute toward the development of the research as a whole (Cohen and Crabtree, 2006). They assist in the examination of certain topics as a result of the communication that moves beyond the verbal, and enables the researcher to draw links between various parts of people's lives, both seen and unseen (Edwards and Holland, 2013). The answers that were given were open-ended, creating more room for the participants to elaborate on certain areas, or points that were raised, which were of particular interest (Roberts et al., 2003). Interviews were transcribed and underwent a thematic analysis. Thematic analysis is frequently used in qualitative research as it examines classifications and existing patterns that relate to the data. In other words, it seeks to provide meaning, precision and intricacy to various aspects of the study at hand (Alhojailan, 2012). It is a valuable research tool as it provides a rich and comprehensive, yet multifaceted interpretation of data (Braun and Clarke, 2006).

The participants in this study are young (18–25) black South African women, who each had, at the time of undertaking the research, over 10,000 Instagram followers (in most cases this has since increased). All had similar online profiles, featuring inspirational quotes alongside images of them wearing fashionable clothing and accessories and posing in attractive ways and desirable locations. All of these women also periodically posted material that seemed more personal and intimate. All appear very well-groomed and pride themselves on paying careful attention to their image. From head-to-toe, they suggest a certain understanding of success. They are engaged in the kind of “aesthetic vigilance” that involves “a calculative and self- governmental labour of risk-managing one's attachments to beauty and its technologies” (Dosekun, 2017, p. 169). During the interviews it became apparent that all of these women are ambitious, believe they are capable of achieving much, and claim that they want to “make something” of themselves, constantly asserting their independence, in line with theoretical ideas about post-feminist practise (Tasker and Negra, 2007).

According to McRobbie, “Black women co-inhabit the space of attention with their white counterparts, but it is always understood that they are exceptional and act as role models for their less successful peers” (2009, p. 132). This speaks to the societal shift that is played out on Instagram, where certain women see their function as not just being beautiful, but also about evaluating every area of their lives and managing how meaningful and impactful they are to other women, in this case their Instagram followers. Authenticity thus becomes an imperative for these young women, not only in gaining attention and status but also in behaving in a way that is inspiring for their following. Performances of authenticity are thus inherently moralised, part of a social imperative by which “exceptional” black women are expected to hold themselves to particular standards.

There are necessarily limits to a study such as this one, which is concerned with a small pool of interviewees who share traits that are relevant to the interviewers' concerns. We do not claim that these women's viewpoints are generalizable to all young black South Africans, to all Instagrammers or even to all local micro-celebrities. Nonetheless, their viewpoints offer us an important entry point to considering some of the ways in which social media intimacy intersects with aspiration and identity in the South African context. Future research could consider how authenticity is perceived and valued among a larger or more varied group of Instagram users in order to enrich these perspectives with comparative discussion. Researchers could also delve more deeply into the histories and inner lives of micro-influencers to see how their personal social experiences impact on their digital selves, or consider the digital experiences of more traditional celebrities.

## Living an Authentic Life Online

According to Tasker and Negra, the “imaginary self,” the self that is performatively constructed, “must not only be discovered, but experienced, paradoxically, as more authentic than the previous one, which comes to be regarded as inauthentic. It is thus through technological shifts that it is believed that one can discover their more authentic true self” (2007, p. 237). These “technological shifts” include the introduction of social media, which facilitate the creation of an online self that is as much a product of imagination, desire and socio-cultural cues as of individuality. While this authenticity may be performative, it seems to be necessary that is *experienced* as legitimate, even while it is intentionally undertaken. During the interviews the participants constantly emphasised their own realness and normality. As Participant I explained, “I am an everyday person”^2^.

These women perform their online identities through the pictures, captions and quotes they post and the overall image they portray, which comes to be “understood as evidence of backstage behavior and a “true” personality [in the] seemingly authentic and socially connected context of online social media” (Ellcessor, 2012, p. 60). Followers' apparently free access to this true personality mirrors the “parasocial interaction” (Marwick, 2015, p. 139) that is common within more traditional celebrity. Followers' comments, and the participants' own evaluations of their brands and desirability, confirm that this kind of realness is a valuable commodity in creating communities and identifications. In a sense, “the authentic has been colonised by calculated self-promotion” (Salisbury and Pooley, 2017, p. 6). Participant F explained, “I'm human, I am allowed to post specific real things.” Participant H said, “The more real I am, the easier it makes it for people to want to know who I am,” a point that was repeated by Participant I, “If I post relevant things, I attract more women that want to feel normal.” Despite the carefully curated glamour of their online aesthetic, they insist that their relatable normality is a significant part of their appeal. In explaining decisions about her online persona, Participant B said, “People caught onto me being real and being myself, so I decided I'm just going to try and be more of that.” The word “try” suggests that there is a conscious effort involved in the way she authenticates her self-display, a claimed normality that makes her more accessible to the average audience member (Wood, 2013). Participant A stated,

It's just showing people I am ordinary. I get a lot of girls inboxing me, and we talk about these sorts of normal things. It's not only about empowering women, it's also about showing people I'm ordinary, I'm living my life to the best of my ability, and basically this is a process of my life. I go to school, I have heartbreaks, and I have bad days and good days. I'm not perfect, I'm Christian, I'm a believer.

Similarly, Participant C explained, “I am a normal person just like everyone else. I'm still as humble as possible, so I think that's what makes me different.” Her claim here centralises authenticity as a primary part of her appeal: she is *still* humble, she remains a “normal” person despite her extraordinary qualities. This version of realness was echoed by Participant D, who explained what she wanted people to say about her: “Wow, she's actually a really nice person, she's just as normal as we are.”

Participants feel there is an expectation for them to be “real” with the people who follow them, and that this realness is as much a part of their role as fostering “empowerment” by modelling their own post-feminist success online. Authenticity is part of a toolkit of strategies that facilitates success on Instagram, that is carefully designed to imply the intersection of achievable normality with aspirational glamour. Indeed, claimed authenticity may be the key to the success of these kinds of micro-celebrities: their lives seem both intensely desirable and actively achievable, notwithstanding how out of reach that level of consumption remains for many of the young black South African women who make up their audience.

The contradiction of performed authenticity again recalls the figure of the neoliberal woman. This understanding of selfhood encourages people to be self-reliant by viewing themselves as autonomous subjects endowed with choices (Brown, 2006), and to “see themselves as individualized and active subjects, responsible for enhancing their own well-being” (Larner, 2000, p. 3). Within a neoliberal social order, individuals are expected to be “entrepreneurs of themselves or… investors in themselves, as human capital that wishes to appreciate” (Feher, 2009, p. 30, 31). This investment can be clearly seen in the way in which Participant F envisioned the rewards of authenticity: “How you reflect yourself from your soul, your personality, the way you present and carry yourself, your morals and your integrity makes you a beautiful woman.” Notwithstanding their emphasis on idealised notions of naturalness and realness, this account makes clear the way in which desirable forms of femininity are archetypal and must be consciously adopted rather than being inherent. As well as using their online self-displays to tell their stories and gain social media capital, these women also suggest that online performance gives them an opportunity to become the “best version” of themselves. Participant D explained, “I try my best to be the best woman I can be all-round, but I make sure not to lose myself in all of that.” This idea ties into neoliberal notions in which women are expected to engage in “self-government, self-discipline and self-management” to better themselves (Gill, 2007b, p. 163, 164), and also highlights the contradiction that becoming one's best online self is not necessarily coherent with being authentic. According to Participant G, “I realised people follow me, and are waiting for my next move.” She is aware of her audience and monitors her activity and actions, positively filtering what she posts and how she portrays herself. Participant B explained, “I know I need to watch what I post. So now I am more cautious about how I conduct my Instagram page.” Even as the appearance of authenticity is prized, the participants engage in constant self-monitoring and self-discipline to ensure that nothing that makes its way into their Instagram feeds will threaten the illusion of unvarnished reality.

Traits that the participants defined as authentic emerged at numerous points in our interactions, and were not always what we would think of as positive. Many admitted that they make mistakes, have regrets and deal with the consequences of their choices, yet they say their followers still refer to them as “inspirational.” These admissions of failure, fault or weakness ranged from acknowledging that they are still carrying post-baby weight or admitting that having a baby was never a part of their plan to the reality that they still use public transport or have days when they do not feel beautiful, regardless of what the outside world may see or say. Participant D said, “As young as I am and as much as I have made mistakes, I am human, and I inspire a lot of people because I am wise, and because of my integrity.” Here she defines her mistakes as part of rather than a threat to her status: her integrity in acknowledging and dealing with these errors adds to rather than undermines her status. Participant F explained, “Having a baby was hard because it was not planned, and I think I surprised a lot of people, as many questioned how a smart girl like me could have fallen pregnant. When I found out, the first thing I said was what are people going to say?” According to her understanding, “smart” women do not get pregnant by accident (Barnett, 2004). This is an experience that is likely to be relatable within the context of South Africa where younger, premarital and single parenthood are relatively common (Kaufman et al., 2001).

Participant F continued,

I could have terminated the baby, and continued being the “it” girl, and probably suffered in the future from the consequences of terminating, just because I was afraid of society. But in the end, it was my decision, I decided to keep it, and I decided that I am going to turn this mistake into a blessing.

Here the participant actively defines her response to this mistake as a positive and inspirational feature, showcasing her authentic individuality in choosing to keep her baby rather than bend to social pressure. She further explained, “I believe I gave people the liberty to feel that even if you have made a mistake, and you are a mom, you can still live, be beautiful and take charge of your body and become a better person through your own choices.” The accidental pregnancy becomes part of her personal myth, allowing her to present herself as an empowering force for the women who look up to her. Once publically admitted in the appropriate tone, these participants' “failures” become part of their success.

## Individuality and Uniqueness

The women in this study have identified Instagram as one of those significant sites “where power is made at various intervals within everyday life” (Butler et al., 2000, p. 14), and have found ways to construct their online identities that allow them to benefit from that power in the form of status and social capital. Words like “inspirational” and “role model,” which appear frequently in their Instagram comments, emphasise their role in shaping identity and ideological building. As Participant H explains, “I always have girls sending me DMs telling me how much they look up to me and how much I inspire them.” They use their ideas of authenticity both to define the kinds of practises they undertake online and to convince their audiences to believe in them.

McRobbie (2009, p. 19) suggests that, under conditions of post-feminism, women are “called to invent their own structures.” These structures are, however, unlikely to be entirely original or entirely free. As suggested above, the women in this study are entangled in a postfeminist paradox: their effective and agential use of the empowering space of Instagram is constrained by structural conditions. In order to succeed on Instagram they monitor themselves and their behavior, and consequently construct themselves and their identities to ally with a commonly understood notion of authenticity. Being “successful on your own terms,” an achievement that is prized by these micro-celebrities, implies being successful in ways that are prescribed by a consumer, capitalist and patriarchal society. The discourses that these women use make a point of highlighting their unique individuality while also suggesting that only a very specific mode of (visible, public, performed) authenticity is legitimate.

During the interviews participants frequently restated the importance of emphasising their individual interests, skills and styles. Many of the participants had active strategies for managing their individuality: “Continuity is key. Pick something and stick to it” (Participant C); “If I love dogs, I will showcase that, and you can get an informed perception about me” (Participant H); “Don't be all over the place. Find something, stay true to it, run with it” (Participant G). There is a potential contradiction lurking here: one must find something that will appeal to an audience, which suggests a degree of artifice, but then must stay true to it, which defines the interest as genuine. The fact that the participants did not experience this as contradictory suggests a convergence between their online and offline identities, an internalisation of their ideas of authenticity.

The participants emphasise the importance of their individuality by mentioning that a successful Instagram personality must “find their own trade and stick to it” (Participant C). However, their profiles all bear strong resemblances to each other in terms of style, design, poses, fashion, clothing, accessories, brands and other aesthetic features. In a neoliberal society, “people are increasingly individualised. They are required to invent themselves, and they are repeatedly called upon to shape themselves so as to be flexible to fit within new circumstances which makes them aspirational” (McRobbie, 2009, p. 130). This need for the appearance of individualisation can help to explain why the participants are so focused on their own uniqueness, even when they often admit—albeit obliquely—the calculations that go into their Instagram success. When asked about her aesthetic similarity to other micro-celebrities, Participant A explained, “They are not me and I am not them. Whatever I post is authentically me.” The participants identify themselves as individuals, comfortable in who they are and secure in their own individuality, determined to be themselves “the best way I can” (Participant B). Looking at their pages it becomes clear that, far from emphasising uniqueness, this form of micro-celebrity depends on one's ability to successfully embody a pre-existing archetype.

The participants also emphasised the fact that Instagram is not really a true depiction: it is only a fragment of reality, and followers only see what they choose to post. The versions of their lives and selves that are available online are heavily mediated (Matic, 2011). Participant B explained, “Instagram is Instagram, and people take Instagram too literally.” They were also aware of the ways in which performances of realness and authenticity actively contribute to their online brands and shield them from certain types of criticism. Participant B continued, “You don't want to walk down the road and for people to say she's such a dream-seller, and in the meantime, you know you are just an ordinary person. So, I try by all means to take photos of what is really happening.” These interviews suggest that, rather than a deeply held sense of subjecthood, being “true to yourself” requires you to be consistent with your portrayed image, the self that people have come to know. It is important that the online and offline selves remains coherent and that one's digital identity appears seamless and genuine, regardless of how much labour goes into it. Participant D explained, “My posts are not superficial. Whenever I post it is what is happening. I can never be questioned about the lifestyle I lead, because it is really the lifestyle I lead, not some façade.” Again, this approach suggests a classed dimension to these postfeminist performances. These women are wary of appearing like frauds or imposters. They continually emphasise that the glamorous lives they display online are legitimate.

The participants' investment in the idea of authenticity extends to the belief that their offline personas match their online display, as Participant E said, “I'm pretty much always the same, online and offline.” This statement suggests that her offline persona is also monitored, and that there is a degree of intentional and performative self-construction at every level of daily life. If we accept their claims about the cohesion between their Instagram profiles and their offline selves, the lives of the participants could be seen as a performance of authentic living, as “performance engulfs a radically constructionist notion, suggesting that personal narrative performance is a site in which social meaning – including that of a narrator's identity – is fervently negotiated and constructed” (Noy, 2004, p. 167). The kind of display that leads to success and micro-celebrity on Instagram must be, at least to some extent, carried through into daily life in order to assure that both identities remain workable. Participant B explained the importance of the online image by mentioning that she does not want people seeing someone different when she is in public: “Aren't you the girl from Insta? Every time I'm going out to a place where there are a lot of people, I make sure I look good so they can't say oh she's a catfish.” In order to match her online identity she makes a conscious effort to always dress well and look good so as to avoid possible dissonance from fans and followers, to the extent of being accused of catfishing: of pretending to be someone entirely different, and completely inauthentic, in order to fool, trap, manipulate and humiliate others. These kinds of claims would be counter to the affect of her authenticity, the desire to be seen as real, normal and “nice.”

The participants also felt that they have a duty to share personal information with their followers. Participant I said, “I try my best to display the actual person I am, my thoughts and the things I go through, because that is what people want. Sometimes I even talk about my mom on my posts. How I act or talk to people is really the same.” Participant E explained, “I just try to keep it real and welcoming by actually displaying the person I am, even outside Instagram.” The humanness and vulnerability these women choose to show, like the personal details they provide, are tools that they use to create their online selves. Participant H said, “I just want my page clean, showing that's me in my normal life. I don't pretend to be someone and something I am not so that I can be accepted.” Participant J explained, “I'm just a normal person 90% of the time. If a person sees me, they will say this is the same person.” What is essential in these accounts is that these women place so much value on being authentic not just for themselves and their own consciences, but for their followers. They are deeply conscious of the opinion of their followers and this has weight in shaping their identities.

## The Pressures of Social Media

The participants generally feel that they are empowered and liberated from societal pressures. Participant G stated, “I don't feel the need to do things just because society says so.” At the same time, however, all confessed to conforming to the norms of Instagram at different times and in different ways, and many offered meaningful insights about how the structures of social media affected their behavior. They explained that “social media really does make you lose your focus” (Participant I) as “you feel the pressure to conform to what others are doing” (Participant A). These norms included going to certain places just to post that they were there and wearing certain things just so they could post a picture for their followers to like. Participant C explained, “You do things without actually realising you're doing it for your followers, you're not doing it for yourself. You will literally buy an outfit and you just want to post wearing it, and then you realise your life is Instagram now. You're no longer living a normal life.” Participant A said, “You begin doing things you do not really want to do just to impress, be loved and recognised.” This tendency has been termed “doing things for the 'Gram” (Participant F). As much as post-feminist discourse suggests that femininity is associated with choice, and as much as these women display genuine agency in making their decisions, the kinds of choices they make are rooted in existing structures. According to Gill (2007b, p. 157), “Women are presented as entirely free agents, but this avoids all the important questions about the relationship between representations and subjectivity, the difficult but crucial questions about how socially constructed, mass mediated ideals of beauty are internalised and made our own.” Participant J described her behavior as coming from a place of enlightenment regarding the choice she feels she has: “It's just another social media platform. Everything I do is for myself, but it is important to be the person I am and show people that. If they don't see it that way, that's their own thing.” This emphasis on genuine, honest choice that is true to each individual's deep desires is complicated by these women's admissions of the body work, surveillance and self-discipline required for Instagram success.

Tasker and Negra (2007, p. 237) argue that “the new self must be liberated, rather than being imposed from the outside. The more authentic self bears the hallmark of post-feminism.” Many of the participants stated that as they grew and matured they began to see what they call the “superficiality” of Instagram, which led them to develop online personas that valued authenticity. Participant G said, “I am who I put out there, I'm not living a lie.” All of the participants made it clear that one can only achieve this liberation from societal expectation when one is confident and has a solid understanding and acceptance of oneself as an individual. Participant D explained, “Your likes don't determine who you are. I got to understand that, and people need to understand that.” Participant I stated, “I'm the type of person that can leave social media.” She defines her sense of self, of the kind of person that she is, by her (untested) freedom to walk away from her online life.

Although the participants' engagement with Instagram and with their followers seems independent and driven by personal choice, the form of their freedom is challenged by the fact that they are expected to “improve” themselves by continually investing in self-maintenance practises. This also brings into question their definition of authenticity: if the self must be constantly disciplined and improved, how can it also be authentically real as it is? This suggests that what is to be considered as authentic is in need of constant monitoring, which disqualifies the common understanding of authentic and again points to authenticity as a performance.

## Conclusion

The young women interviewed for this project make it clear that ideas of authenticity and realness influence their self-display and sense of self-worth on Instagram and in real life. However, the way in which the participants define, understand and live out their authenticity or realness appears different to the commonly held understanding of what it means to be real and authentic. Certain habits, such as the adoption of constant self-modification techniques, have come to be a part of their daily practise. The authenticity that is prized by these micro-celebrities and their followers seems akin to a performance in which apparent realness must be displayed and highlighted, in contradiction to understandings of realness that locate it within individual humanness.

This aspirational interpretation of authenticity requires that these women emphasise their normality and relatability while at the same time maintaining stringent standards in terms of the aesthetics of their bodies, appearances, clothing and Instagram personas. The interviews revealed the importance of Instagram in allowing them to display their individuality, their mistakes, their personal struggles and their life events in order to represent themselves as normal, everyday individuals. This highlighted the way that they go about constructing every aspect of their identities in the attempt to become who they aspire to be and who they feel they are expected to be, and as a result of this, emerge as being inspiring to others. Authenticity, while highly valued, is treated as part of a larger toolkit that allows for success on Instagram and consequently in offline life, as these micro-celebrities' aspirational status and cultural capital impacts on their social worlds.

In acknowledging the bodily and psychic self-discipline needed to succeed in the competitive world of Instagram alongside the more affective requirement of appearing authentic, these women illustrate some of the paucities of post-feminist praxis, which promises freedom, agency, power and selfhood – but only if one does the right sort of work first.

## Data Availability Statement

The dataset presented in this article is not readily available. Interview recordings and transcripts were anonymised and stored by the researcher and will be destroyed after a certain time period, in line with University ethics committee norms. Requests to access the dataset should be directed to Nicky Falkof, nicky.falkof@wits.ac.za.

## Ethics Statement

This study was approved by Postgraduate Ethics Committee of the Faculty of Humanities, University of the Witwatersrand. The participants provided written informed consent to participate in this study.

## Author Contributions

The article is based on graduate work undertaken by CD, under the supervision of NF. Both authors contributed to the article and approved the submitted version.

## Conflict of Interest

The authors declare that the research was conducted in the absence of any commercial or financial relationships that could be construed as a potential conflict of interest.

## Footnotes

^1^This article is drawn from a longer study that combined interviews with visual discourse analysis of Instagram posts to consider the linked themes of authenticity, celebrity and beauty within these young women's Instagram identities (Dunn, 2017).

^2^Interview participants have been anonymised and assigned letters from A to J in order to differentiate them in the discussion that follows.
